# Impact of Excessive Increase in Systolic Blood Pressure after Exercise on Clinical Outcomes in Patients with ST-Segment Elevation Myocardial Infarction

**DOI:** 10.3390/jcm12216928

**Published:** 2023-11-04

**Authors:** Takahiro Yamashita, Kenichi Sakakura, Hiroyuki Jinnouchi, Yousuke Taniguchi, Takunori Tsukui, Masashi Hatori, Yusuke Tamanaha, Taku Kasahara, Yusuke Watanabe, Kei Yamamoto, Masaru Seguchi, Hideo Fujita

**Affiliations:** Division of Cardiovascular Medicine, Saitama Medical Center, Jichi Medical University, 1-847 Amanuma, Omiya, Saitama City 330-8503, Japan

**Keywords:** acute myocardial infarction, ST-elevation myocardial infarction, 200 m walk test, cardiac rehabilitation, increased SBP, outcomes

## Abstract

Objective: Although the clinical outcomes for patients with ST-elevation myocardial infarction (STEMI) have improved significantly, some patients still experience poor clinical outcomes. The available risk classifications focus on the short-term outcomes, and it remains important to find high-risk features among patients with STEMI. In Japan, the 200 m walk electrocardiogram (ECG) test is widely performed before discharge. The purpose of this study was to investigate the association between the excessive increase in systolic blood pressure (SBP) following a 200 m walk and the long-term clinical outcomes in patients with STEMI. Methods: We included 680 patients with STEMI and divided those into an excessive increase in SBP group (*n* = 144) and a non-excessive increase in SBP group (*n* = 536) according to the SBP increase after a 200 m walk ECG test. We defined an excessive increase in SBP as SBP ≥ 20 mmHg either just after or 3 min after a 200 m walk ECG test. The primary endpoint consisted of major cardiovascular events (MACE), defined as the composite of all-cause death, non-fatal myocardial infarction, readmission for heart failure, and ischemia-driven target vessel revascularization. Results: The median follow-up duration was 831 days. MACE was more frequently observed in the excessive increase in SBP group (24.3%) than in the non-excessive increase in SBP group (15.1%). Multivariate Cox hazard analysis revealed that the excessive increase in SBP was significantly associated with MACE (HR 1.509, 95% CI: 1.005–2.267, *p* = 0.047) after controlling for multiple confounding factors. Conclusion: An excessive increase in SBP after the 200 m walk ECG test was significantly associated with MACE in patients with STEMI. The 200 m walk ECG test is simple and low-cost, but may help to identify high-risk patients with STEMI.

## 1. Introduction

The clinical outcomes of patients with ST-elevation myocardial infarction (STEMI) have improved significantly through the spread of primary percutaneous coronary intervention (PCI), intensive care units, and optimal medical therapy [1,2]. However, some patients still experience poor clinical outcomes [3]. Although some patients with STEMI have been classified as high-risk according to well-known risk scores such as the Global Registry of Acute Coronary Events (GRACE) score or the Thrombolysis in Myocardial Infarction (TIMI) risk score, the available classifications focus on the short-term outcomes rather than the long-term outcomes [4,5,6]. Therefore, it is still important to find high-risk features for the improvement of mid-term or long-term clinical outcomes in patients with STEMI. 

In Japan, the 200 m (and 500 m) walk electrocardiogram (ECG) tests are widely performed during hospital stays for acute myocardial infarction (AMI) [7]. The primary purpose of these ECG tests is to identify patients with residual ischemia prior to hospital discharge. However, a slow gait speed during 200 m walk test is also reported to be associated with an increased risk of cardiovascular events [8]. Although the excessive increase in blood pressure is one of the criteria for unsuccessful 200 m (and 500 m) walk ECG tests [7,9], it remains unclear whether an excessive increase in blood pressure after a 200 m walk has an impact on long-term outcomes in patients with STEMI. The purpose of this retrospective study was to investigate the association between the excessive increase in blood pressure following the 200 m walk test and the long-term clinical outcomes in patients with STEMI.

## 2. Methods

We reviewed all patients with AMI who were treated at our institution (Saitama Medical Center, Jichi Medical University) between January 2015 and December 2021. The inclusion criterion was (1) patients with AMI. The exclusion criteria were as follows: (1) patients with non-ST elevation myocardial infarction; (2) patients who did not undergo primary PCI for the culprit lesion; (3) patients who underwent cardiac surgery, such as CABG; (4) patients who could not walk the 200 m distance during index hospitalization; (5) patients who had missing data on blood pressure levels before and after the 200 m ECG test; and (6) a second or more than second STEMI during the study period, i.e., when a patient experienced ≥2 STEMI during the study period. 

The 200 m walk ECG tests were performed by registered nurses in general cardiology wards. Patients were instructed to walk 2 laps down the 50 m corridor in general cardiology wards at their own pace. Patients wore an ECG monitor during the walk. The 12-leads ECG and vital signs were registered by nurses at 3 points (before the test, just after the test, and 3 min after the test). We defined an excessive increase in systolic blood pressure (SBP) as an increase in the SBP to ≥20 mmHg just after or 3 min after 200 m walk ECG test. The patients involved in the study were divided into an excessive increase in SBP group and a non-excessive increase in SBP group according to the SBP increase just after or 3 min after the 200 m walk ECG test. The primary endpoint was a major cardiovascular event (MACE), defined as the composite of all-cause death, non-fatal myocardial infarction, readmission for heart failure, and ischemia-driven target vessel revascularization. Information regarding the above clinical outcomes was acquired from hospital records. The day of the 200 m walk ECG test was defined as the index day (day 1). The patients were followed until the point of MACE or until the study’s end date (26 December 2022). This study was approved by the institutional review board of the Saitama Medical Center, Jichi Medical University (S23-036), and written informed consent was waived because of the retrospective study design. The data collection and storage were performed anonymously, according to the guidelines of the Japan Ministry of Health, Labour, and Welfare.

### 2.1. Definitions

AMI was defined according to the universal definition [10]. Diagnostic ST elevation was defined as new ST elevation at the J point in at least two contiguous leads of 2 mm (0.2 mV), and the AMI patients with ST elevation were diagnosed as STEMI [11,12]. The definitions of hypertension, diabetes mellitus, and dyslipidemia were described elsewhere [13,14]. We used the laboratory data recorded at admission. Since we were unable to measure some laboratory data, such as HbA1c and low-density lipoprotein (LDL) cholesterol levels, during off hours (night or holidays), we substituted the earliest HbA1c or LDL cholesterol levels recorded since admission for the laboratory data [15]. Left ventricular ejection fraction (LVEF) was measured by transthoracic echocardiography during the index hospitalization. LVEF was calculated through either the modified Simpson’s method, the Teichholz method, or eyeball estimation [16]. The Teichholz method was adopted only when the modified Simpson’s method was not available. An eyeball estimation was adopted only when both the modified Simpson’s method and the Teichholz method were unavailable. We also calculated the estimated glomerular filtration rate (eGFR) using serum creatinine (Cr), age, weight, and gender according to the following formula: eGFR = 194 × Cr^−1.094^ × age^−0.287^ (male), or eGFR = 194 × Cr^−1.094^ × age^−0.287^ × 0.739 (female). The initial thrombolysis in myocardial infarction (TIMI) flow grade and final TIMI flow grade were recorded according to coronary angiography [17]. 

### 2.2. Statistical Analysis

The Shapiro–Wilk test was performed to determine whether the continuous variables were normally distributed or not. Data are presented as percentages for categorical variables, means  ±  standard deviations (SDs) for normally distributed continuous variables, and medians (quartile 1–quartile 3) for nonparametric variables. Categorical variables are presented as numbers (percentage), and were compared using the Chi-square test. Normally distributed continuous variables were compared using the Student t-test. Otherwise, continuous variables were compared using the Mann–Whitney U test. Event-free survival curves were constructed using the Kaplan–Meier method, and the statistical differences between curves were assessed by the log-rank test. We also performed a multivariate Cox hazard analysis to investigate the association between excessive increases in SBP and MACE after controlling for confounding factors. In this model, MACE was adopted as a dependent variable. Variables that were significantly different (*p* < 0.05) between the excessive increase in SBP group and the non-excessive increase in SBP group were included as independent variables in the model. The hazard ratios and 95% confidence intervals (CI) were calculated. A *p* value < 0.05 was considered statistically significant. All analyses were performed using statistical software, i.e., SPSS 25/Windows (SPSS, Chicago, IL, USA).

## 3. Results

**Figure 1:**
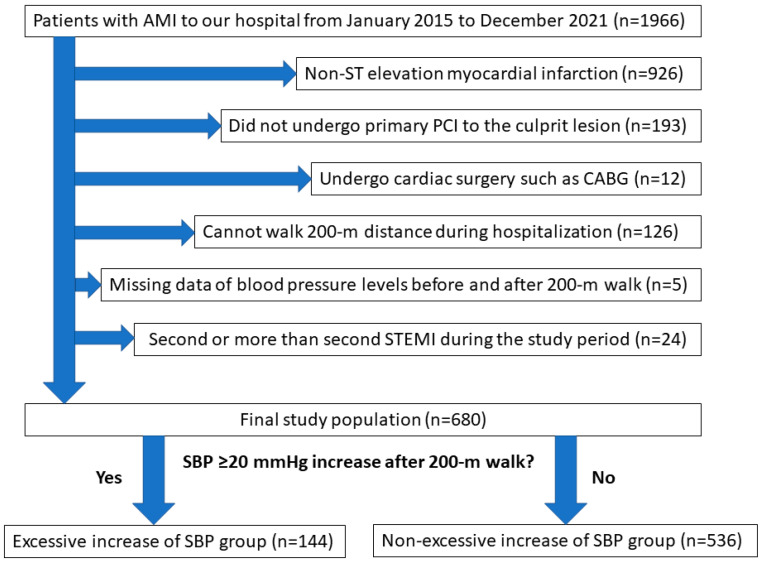
Study flowchart. Abbreviations: AMI = acute myocardial infarction, PCI = percutaneous coronary intervention, CABG = coronary artery bypass graft surgery, SBP = systolic blood pressure.

From January 2015 to December 2021, a total of 1966 patients with AMI were admitted to our medical center. After excluding 1286 patients who met the exclusion criteria, the final study population consisted of 680 patients with STEMI. They were divided into an excessive increase in SBP group (*n* = 144) and a non-excessive increase in SBP group (*n* = 536). The study’s flow chart is shown in Figure 1. 

A comparison of characteristics between the patients in the two groups is shown in Table 1. The median age was significantly older in the excessive increase in SBP group than in the non-excessive increase in SBP group. The peak creatine kinase (CK) and the creatine kinase–myocardial band (CK-MB) were significantly lower in the excessive increase in SBP group than in the non-excessive increase in SBP group. BNP was significantly higher in the excessive increase in SBP group than in the non-excessive increase in SBP group. Diuretics were more frequently prescribed on the day of the 200 m walk ECG test in the excessive increase in SBP group than in the non-excessive increase in SBP group. The SBP before the 200 m walk ECG test was similarly controlled between the two groups.

Table 2 shows the comparison of lesion and procedural findings between the two groups. The characteristics of the lesions were similar between the two groups. Non-invasive positive pressure ventilation (NPPV) was more frequently used in the excessive increase in SBP group than in the non-excessive increase in SBP group. The other procedural characteristics were not different between the two groups.

**Figure 2:**
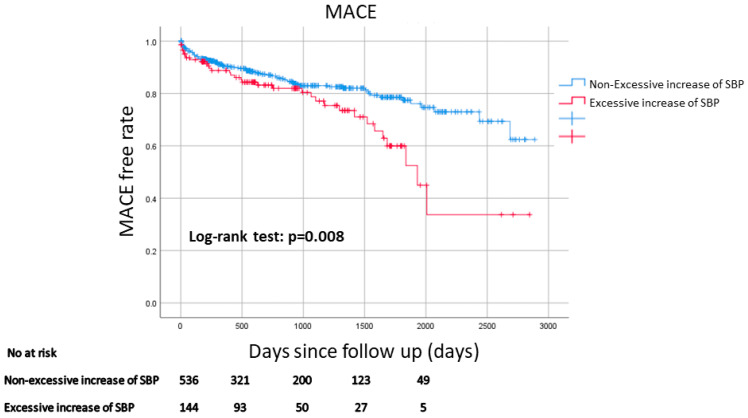
Kaplan–Meier curves for MACE-free survival between the excessive increase in SBP group and the non-excessive increase in SBP group. A log-rank test was used. Abbreviations: MACE = major cardiovascular events.

Table 3 shows a comparison of the clinical outcomes between the two groups. The median follow-up duration was 831 days (Q1: 263 days–Q3: 1530 days). Figure 2 shows the Kaplan–Meier curves for MACE-free survival between the two groups. MACE was more frequently observed in the excessive increase in SBP group than in the non-excessive increase in SBP group. The multivariate Cox hazard analysis is shown in Table 4. An excessive increase in SBP was significantly associated with MACE (HR 1.509, 95% CI: 1.005–2.267, *p* = 0.047) after controlling for age, eGFR, peak CK, use of diuretics on the day of the 200 m walk ECG test, and use of NPPV. 

## 4. Discussion

The main findings of this study are as follows: (1) We followed up on 680 patients with STEMI after hospital discharge with a median duration of 831 days, and found that MACEs were more frequently observed in the excessive increase in SBP group than in the non-excessive increase in SBP group, and (2) the multivariate Cox hazard analysis revealed that an excessive increase in SBP after the 200 m walk test was significantly associated with MACE after controlling for multiple confounding factors. Our results suggest that the excessive increase in SBP may be one of predictors of poor clinical outcomes in patients with STEMI.

Specific literature regarding the 200 m walk ECG test in patients with AMI is sparse, but there are several studies regarding the blood pressure response after stress tests in patients with AMI or coronary artery disease. Kato et al. conducted a treadmill exercise test for 217 patients with AMI at an average of 9.3 weeks after AMI, and defined an abnormal post-exercise SBP response as a ratio of SBP at 3 min of recovery to peak exercise of 0.9 or more [18]. An abnormal post-exercise SBP response was significantly associated with cardiac death [18]. This finding was in line with our results, although we focused on long-term outcomes. Huang et al. included 3054 patients with suspected coronary artery disease who underwent exercise treadmill tests, and they defined a paradoxical SBP increase as an SBP at 3 min of recovery equal to or higher than that at 1 min of recovery [19]. They found that the combination of an ischemic ST-segment change and a paradoxical SBP increase were associated with long-term mortality [19]. Although they adopted a combination of ischemic ECG changes and an SBP increase, we adopted an SBP increase alone, because an ischemic ST-change after exercise is an established risk factor for clinical outcomes. Hashimoto et al. investigated the mechanism of abnormal SBP response during exercise recovery in patients with angina pectoris [20]. Their finding was also in line with our results. However, their study population consisted of patients with angina pectoris, whereas our study population comprised patients with STEMI. They inserted a Swan–Ganz catheter during bicycle ergometric studies, and concluded that an abnormal SBP response after exercise is indicative of severe myocardial ischemia and may be caused by an increase in stroke volume due to recovery from myocardial ischemia [20]. However, these studies were conducted before the development of drug-eluting stents or primary PCI, while all patients in our study underwent primary PCI for the culprit lesion of STEMI. Furthermore, the energy intensity for the 200 m walk test at the patient’s own pace is supposed to be mild (2–3 METs). The meaning of an excessive increase in SBP after 200 m walk ECG test would be different from that of the SBP response after a treadmill exercise test. 

We should discuss why the excessive increase in SBP after the 200 m walk test was associated with long-term MACE. A possible explanation is increased arterial stiffness. Sung et al. reported an association between a higher SBP during exercise treadmill test and high brachial-ankle pulse wave velocity, which is an indicator of arterial stiffness, in normotensive persons [21]. Although the baseline SBP prior to the 200 m walk test was similar between the two groups, the mean age was significantly higher in the excessive increase in SBP group than in the non-excessive increase in SBP group. Advanced age is strongly associated with increased arterial stiffness [22]. Another mechanism is the baroreflex failure, which results in excessive fluctuations of arterial blood pressure [23]. Veronese et al. reported that orthostatic hypertension was a predictor of all-cause mortality in elderly patients [24]. Therefore, arterial stiffness and baroreflex failure might be possible reasons for the association between the excessive increase in SBP after a 200 m walk test and long-term MACE in patients with STEMI.

There are several potential confounding factors, including age, eGFR, BNP, peak CK, use of diuretics, and the need for NPPV. Although all these factors, except for BNP, were included as variables in our multivariate Cox hazard analysis, we should discuss the association of each factor with excessive SBP increase. As mentioned in the previous paragraph, age is strongly associated with increased arterial stiffness [22], which results in excessive SBP change. Estimated GFR is inversely associated with age, because the calculating formula for eGFR includes age. Higher levels of BNP, use of diuretics, and the need for NPPV were more frequently observed in the excessive increase in SBP group than in the non-excessive increase in SBP group, which suggests the presence of heart failure as a potential reason for the excessive increase in SBP. Interestingly, peak CK levels were lower in the excessive increase in SBP group than in the non-excessive increase in SBP group. These findings suggest that an excessive increase in SBP is not associated with myocardial damage caused by STEMI, but with the presence of heart failure. 

The clinical implications of the present study should be noted. Although the 200 m walk ECG test is recommended for patients with AMI by the Japanese Circulation Society [7], there have been few studies to support the usefulness of the 200 m walk ECG test [8]. In comparison with traditional treadmill ECG tests, the 200 m walk ECG test does not require special equipment. Furthermore, since a 200 m walk at the patient’s own pace is equivalent to daily physical activity, the risk associated with the 200 m walk ECG test is minimal. We can identify a high-risk group according to information on SBP during a 200 m walk ECG test without additional cost. A careful follow-up may be required for those high-risk patients who show excessive increases in SBP after 200 m walk test. Our findings cannot provide sufficient evidence to change clinical practice beyond current guidelines. The main limitation is that the 200 m walk ECG test is not widely performed after AMI, except in Japan. Our study may become a springboard for the development of the 200 m walk ECG test after AMI outside of Japan. 

Several limitations of this study need to be mentioned. As this study was a single-center, retrospective study, there is a potential selection bias. Each patient underwent the 200 m walk ECG test at a different time. The timing of the 200 m walk ECG test may have led to bias, while the medications, apart from diuretics, were similar at the time of the 200 m walk ECG test between the two groups. Although we identified the risk of an excessive increase in SBP after a 200 m walk ECG test, it is uncertain whether we should administer antihypertensive medications to patients who show excessive increases in SBP. Furthermore, we excluded patients who could not walk a 200 m distance or who died before the day of the 200 m walk test. In other words, we might have excluded the most severe STEMI patients from this study. Finally, ambulatory blood pressure monitoring, which can provide useful information regarding blood pressure variability, was not conducted in this study. 

## 5. Conclusions

An excessive increase in SBP after 200 m walk ECG test was significantly associated with MACE after hospital discharge in patients with STEMI. The 200 m walk ECG test is simple and low-cost, but may help to identify high-risk groups among patients with STEMI. 

## Figures and Tables

**Table 1:** Comparison of patient’s characteristics between the excessive increase in SBP group and the non-excessive increase in SBP group.

	All	Non-Excessive Increase in SBP (*n* = 536)	Excessive Increase in SBP	*p*-Value
	(*n* = 680)		(*n* = 144)	
Age, years	69.0 (60.0–78.0)	68.5 (58.0–77.0)	74.0 (66.3–82.0)	<0.001
Male, *n* (%)	543 (79.9)	435 (81.2)	108 (75.0)	0.103
Body mass index (kg/m^2^)	23.8 (21.8–26.3)	23.8 (21.8–26.3)	23.7 (21.5–25.9)	0.547
Comorbidities				
Hypertension, *n* (%)	520 (76.5)	406 (75.7)	114 (79.2)	0.439
Diabetes mellitus, *n* (%)	267 (39.3)	211 (39.4)	56 (38.9)	>0.999
Dyslipidemia, *n* (%)	364 (53.5)	284 (53.0)	80 (55.6)	0.638
Current smoker, *n* (%)	269/676 (39.8)	220/532 (41.4)	49/144 (34.0)	0.125
Hemodialysis, *n* (%)	27 (4.0)	18 (3.4)	9 (6.3)	0.146
History of previous PCI, *n* (%)	73 (10.7)	55 (10.3)	18 (12.5)	0.449
History of previous CABG, *n* (%)	10 (1.5)	8 (1.5)	2 (1.4)	>0.999
History of previous MI, *n* (%)	54 (7.9)	40 (7.5)	14 (9.7)	0.386
Cardiac arrest at prehospital or ER, *n* (%)	28 (4.1)	25 (4.7)	3 (2.1)	0.236
Shock vital at prehospital or ER, *n* (%)	61 (9.0)	46 (8.6)	15 (10.4)	0.512
Killip class				0.397
1 or 2, *n* (%)	584 (85.9)	462 (86.2)	122 (84.7)	
	33 (4.9)	23 (4.3)	10 (6.9)	
3, *n* (%)	63 (9.3)	51 (9.5)	12 (8.3)	
				
4, *n* (%)				
Region of infarction				0.352
Anterior, *n* (%)	346 (50.9)	271 (50.6)	75 (52.1)	
Inferior, *n* (%)	276 (40.6)	215 (40.1)	61 (42.4)	
Posterior, *n* (%)	58 (8.5)	50 (9.3)	8 (5.6)	
Vital signs				
SBP at admission (mmHg)	140.0 (120.0–162.0)	140.0 (119.0–161.0)	140.0 (122.5–166.0)	0.386
DBP at admission (mmHg)	82.0 (70.3–96.0)	82.0 (71.0–97.0)	80.0 (70.0–95.8)	0.237
Heart rate at admission (beats per minute)	76.0 (63.0–93.0)	76.0 (64.0–92.0)	78.0 (62.3–98.0)	0.683
Laboratory data at admission				
Serum creatinine (mg/dL)	0.82 (0.67–1.01)	0.82 (0.67–1.00)	0.85 (0.68–1.02)	0.383
Estimated GFR (mL/min/1.73 m^2^)	68.6 (54.0–83.1)	69.3 (54.6–84.4)	65.4 (47.5–79.6)	0.037
Hemoglobin (g/dL)	13.9 (12.7–15.2)	13.9 (12.8–15.3)	13.9 (12.2–15.0)	0.058
Platelets (×10^4^/µL)	22.6 (18.7–26.8)	22.7 (18.8–26.6)	21.9 (18.6–27.1)	0.662
C-reactive protein (mg/dL)	0.19 (0.09–0.53)	0.18 (0.09–0.49)	0.24 (0.09–0.99)	0.122
Brain natriuretic peptide (pg/mL)	67.3 (23.1–228.8) (*n* = 658)	59.6 (20.9–182.7) (*n* = 518)	116.5 (36.5–346.7) (*n* = 140)	<0.001
Peak creatine kinase (U/L)	1722 (717–3191)	1788 (777–3253)	1360 (452–2735)	0.018
Peak creatine kinase-myocardial band (U/L)	164.5 (60.3–318)	173.5 (72.0–320.5)	135.5 (34.3–306.0)	0.021
Left ventricular ejection fraction (%)	54.1 (44.8–62.0)	54.0 (45.2–61.9)	54.5 (42.0–62.0)	0.673
Medication on 200 m walk day				
Aspirin, *n* (%)	678 (99.7)	535 (99.8)	143 (99.3)	0.379
Thienopyridine, *n* (%)	669 (98.4)	527 (98.3)	142 (98.6)	>0.999
Beta-blocker, *n* (%)	657 (96.6)	517 (96.5)	140 (97.2)	0.799
ACE-inhibitor, ARB, *n* (%)	659 (96.9)	522 (97.4)	137 (95.1)	0.177
Calcium channel blocker, *n* (%)	46 (6.8)	36 (6.7)	10 (6.9)	0.854
Statin, *n* (%)	673 (99.0)	531 (99.1)	142 (98.6)	0.643
Diuretic, *n* (%)	155 (22.8)	112 (20.9)	43 (29.9)	0.025
Loop diuretic, *n* (%)	112 (16.5)	81 (15.1)	31 (21.5)	0.076
MRA, *n* (%)	74 (10.9)	57 (10.6)	17 (11.8)	0.654
VRA, *n* (%)	7 (1.0)	5 (0.9)	2 (1.4)	0.643
Thiazide diuretic, *n* (%)	5 (0.7)	3 (0.6)	2 (1.4)	0.287
Hypoglycemic agents, *n* (%)	140 (20.6)	111 (20.7)	29 (20.1)	>0.999
Insulin, *n* (%)	24 (3.5)	17 (3.2)	7 (4.9)	0.315
Direct oral anticoagulants, *n* (%)	37 (5.4)	27 (5.0)	10 (6.9)	0.407
Warfarin, *n* (%)	12 (1.8)	11 (2.1)	1 (0.7)	0.477
200 m distance walk				
Pre SBP (mmHg)	112.5 (102.0–124.0)	113.0 (103.0–123.8)	110.0 (100.0–124.0)	0.237
Post SBP (mmHg)	122.0 (112.0–135.0)	118.0 (110.0–130.0)	137.0 (126.3–149.8)	<0.001
Pre HR (bpm)	72.0 (64.0–80.0)	72.0 (64.0–81.0)	70.0 (63.0–78.8)	0.082
Post HR (bpm)	76.0 (67.0–85.0)	75.5 (67.3–85.0)	76.0 (67.0–86.0)	0.622

Data are expressed as the mean ± SD or number (percentage). A Student’s *t* test was used for normally distributed continuous variables, a Mann–Whitney U test was used for abnormally distributed continuous variables, and a chi-square test was used for categorical variables. Abbreviations: PCI = percutaneous coronary intervention; CABG = coronary artery bypass grafting; MI = myocardial infarction; ER = emergency room; GFR = glomerular filtration rate; SBP = systolic blood pressure; DBP = diastolic blood pressure; ACE = angiotensin-converting enzyme; ARB = angiotensin II receptor blocker; MRA = mineralocorticoid receptor antagonist; VRA = vasopressin receptor antagonist.

**Table 2:** Comparison of lesion and procedural characteristics between the excessive increase in SBP group and the non-excessive increase in SBP group.

	All (*n* = 680)	Non-Excessive Increase in SBP (*n* = 536)	Excessive Increase in SBP (*n* = 144)	*p*-Value
Angiographic lesion characteristics				
Culprit lesion				0.664
Left main–left anterior descending artery, *n* (%)	346 (50.9)	271 (50.6)	75 (52.1)	
Right coronary artery, *n* (%)	265 (39.0)	207 (38.6)	58 (40.3)	
Left circumflex, *n* (%)	67 (9.9)	56 (10.4)	11 (7.6)	
Graft, *n* (%)	2 (0.3)	2 (0.4)	0	
Number of narrowed coronary arteries 1 vessel disease, *n* (%) 2 vessel disease, *n* (%) 3 vessel disease, *n* (%)	355 (52.2) 207 (30.4) 118 (17.4)	276 (51.5) 166 (31.0) 94 (17.5)	79 (54.9) 41 (28.5) 24 (16.7)	0.768
50% > stenosis at left main, *n* (%)	45 (6.6)	35 (6.5)	10 (6.9)	0.851
Initial TIMI flow grade of culprit 0, *n* (%) 1, *n* (%) 2, *n* (%) 3, *n* (%)	395 (58.1) 57 (8.4) 115 (16.9) 113 (16.6)	321 (59.9) 42 (7.8) 87 (16.2) 86 (16.0)	74 (51.4) 15 (10.4) 28 (19.4) 27 (18.8)	0.320
Final TIMI flow grade of culprit 0, *n* (%) 1, *n* (%) 2, *n* (%) 3, *n* (%)	2 (0.3) 5 (0.7) 29 (4.3) 644 (94.7)	1 (0.2) 4 (0.7) 24 (4.5) 507 (94.6)	1 (0.7) 1 (0.7) 5 (3.5) 137 (95.1)	0.736
CTO in non-culprit arteries, *n* (%)	68 (10.0)	52 (9.7)	16 (11.1)	0.639
Procedural characteristics				
Mechanical circulatory support devices IABP, *n* (%) Impella, *n* (%) V-A ECMO, *n* (%) Temporary pacemaker, *n* (%)	50 (7.4) 2 (0.3) 8 (1.2) 57 (8.4)	38 (7.1) 2 (0.4) 8 (1.5) 45 (8.4)	12 (8.3) 0 0 12 (8.3)	0.592 >0.999 0.214 1.000
Medical resource use NPPV, *n* (%) Mechanical ventilation, *n* (%)	49 (7.2) 36 (5.3)	29 (5.4) 30 (5.6)	20 (13.9) 6 (4.2)	0.002 0.675
Use of aspiration catheter, *n* (%)	154 (22.6)	122 (22.8)	32 (22.2)	1.000
Final PCI procedure Plain old balloon angioplasty, *n* (%) Aspiration only, *n* (%) Drug coating balloon angioplasty, *n* (%) Bare metal stent, *n* (%) Drug-eluting stent, *n* (%) POBA and aspiration, *n* (%) Other, *n* (%)	23 (3.4) 6 (0.9) 15 (2.2) 8 (1.2) 622 (91.5) 5 (0.7) 1 (0.1)	20 (3.7) 4 (0.7) 12 (2.2) 8 (1.5) 486 (90.7) 5 (0.9) 1 (0.2)	3 (2.1) 2 (1.4) 3 (2.1) 0 136 (94.4) 0 0	0.494
Approach site Trans-radial coronary intervention, *n* (%) Trans-brachial coronary intervention, *n* (%) Trans-femoral coronary intervention, *n* (%)	496 (72.9) 4 (0.6) 180 (26.5)	399 (74.4) 3 (0.6) 134 (25.0)	97 (67.4) 1 (0.7) 46 (31.9)	0.237
Guide-catheter size (Fr) 6, *n* (%) 7, *n* (%) 8, *n* (%)	533 (78.4) 144 (21.2) 3 (0.4)	423 (78.9) 110 (20.5) 3 (0.6)	110 (76.4) 34 (23.6) 0	0.494

Data are expressed as the mean ± SD or number (percentage). A Student’s *t* test was used for normally distributed continuous variables, a Mann–Whitney U test was used for abnormally distributed continuous variables, and a chi-square test was used for categorical variables. Abbreviations: TIMI = thrombolysis in myocardial infarction; IVUS = intravascular ultrasound; OCT/OFDI = optical coherence tomography/optical frequency domain imaging; V-A ECMO = veno-arterial extra-corporeal membranous oxygenation; NPPV = non-invasive positive pressure ventilation; POBA = plain old balloon angioplasty.

**Table 3:** Comparison of clinical outcomes between the excessive increase in SBP group and the non-excessive increase in SBP group.

	All (*n* = 680)	Non-Excessive Increase in SBP (*n* = 536)	Excessive Increase in SBP (*n* = 144)	*p*-Value
MACE, *n* (%)	116 (17.1)	81 (15.1)	35 (24.3)	0.012
All-cause death, *n* (%)	37 (5.4)	24 (4.5)	13 (9.0)	0.039
Non-fatal myocardial infarction, *n* (%)	38 (5.6)	28 (5.2)	10 (6.9)	0.417
Re-admission for heart failure, *n* (%)	41 (6.0)	28 (5.2)	13 (9.0)	0.112
Ischemia-driven target vessel revascularization, *n* (%)	39 (5.7)	29 (5.4)	10 (6.9)	0.544

Data are expressed as numbers (percentages). A chi-square test was used for categorical variables. Abbreviations: MACE = major adverse cardiovascular events.

**Table 4:** Univariate and multivariate Cox hazard analysis to predict MACE.

	Univariate			Multivariate		
Dependent Variable	Hazard Ratio	95% Confidence Interval	*p*-Value	Hazard Ratio	95% Confidence Interval	*p*-Value
Age	1.025	1.008–1.041	0.004	1.015	0.998–1.033	0.084
eGFR at admission	0.982	0.975–0.990	<0.001	0.985	0.978–0.993	<0.001
Peak creatine kinase	1.009	0.921–1.106	0.842	1.067	0.979–1.163	0.141
Use of diuretic on the day of 200 m walk ECG test	1.225	0.773–1.942	0.388	0.750	0.468–1.203	0.233
Use of NPPV	2.575	1.366–4.854	0.003	1.794	1.002–3.210	0.049
SBP increase ≥ 20 mmHg	1.804	1.152–2.824	0.010	1.509	1.005–2.267	0.047

Abbreviations: GFR = glomerular filtration rate; NPPV = non-invasive positive pressure ventilation; SBP = systolic blood pressure.

## Data Availability

The data that support the findings of this study are available from the corresponding author upon reasonable request.
